# Phytoremediation and Microorganisms-Assisted Phytoremediation of Mercury-Contaminated Soils: Challenges and Perspectives

**DOI:** 10.3390/ijerph18052435

**Published:** 2021-03-02

**Authors:** Emanuela D. Tiodar, Cristina L. Văcar, Dorina Podar

**Affiliations:** 1 Department of Molecular Biology and Biotechnology, Babeş-Bolyai University, 1 Kogălniceanu St., 400084 Cluj-Napoca, Romania; dana.tiodar@ubbcluj.ro (E.D.T.); cristina.vacar@ubbcluj.ro (C.L.V.); 2 Centre for Systems Biology, Biodiversity and Bioresources (3B), Babeş-Bolyai University, 3-5 Clinicilor St., 400015 Cluj-Napoca, Romania

**Keywords:** mercury, microbe-assisted phytoremediation, heavy metals, Hg reduction, plants, Hg hyperaccumulator, phytovolatilization, metal sequestration

## Abstract

Mercury (Hg) pollution is a global threat to human and environmental health because of its toxicity, mobility and long-term persistence. Although costly engineering-based technologies can be used to treat heavily Hg-contaminated areas, they are not suitable for decontaminating agricultural or extensively-polluted soils. Emerging phyto- and bioremediation strategies for decontaminating Hg-polluted soils generally involve low investment, simple operation, and *in situ* application, and they are less destructive for the ecosystem. Current understanding of the uptake, translocation and sequestration of Hg in plants is reviewed to highlight new avenues for exploration in phytoremediation research, and different phytoremediation strategies (phytostabilization, phytoextraction and phytovolatilization) are discussed. Research aimed at identifying suitable plant species and associated-microorganisms for use in phytoremediation of Hg-contaminated soils is also surveyed. Investigation into the potential use of transgenic plants in Hg-phytoremediation is described. Recent research on exploiting the beneficial interactions between plants and microorganisms (bacteria and fungi) that are Hg-resistant and secrete plant growth promoting compounds is reviewed. We highlight areas where more research is required into the effective use of phytoremediation on Hg-contaminated sites, and conclude that the approaches it offers provide considerable potential for the future.

## 1. Introduction

Mercury release in the environment has received considerable attention from researchers and legal entities due to its detrimental effects on human and environmental health. Awareness of its toxicity broadened in the second half of the last century, with the Minamata tragedy. Moreover, based on its frequency, toxicity, and potential for human exposure, mercury (Hg) has been ranked 3rd in the Substance Priority List revised by the Agency for Toxic Substances and Disease Registry [1].

In biological systems, Hg is a non-essential element; therefore, usually, there are no dedicated pathways for its metabolization and/or excretion. As a consequence, Hg eventually bioaccumulates and biomagnifies through the food chain [2,3,4,5]. These processes are even more pronounced in the aquatic environment, where certain naturally-occurring microorganisms shift Hg speciation to methylmercury (MeHg), which is easily absorbed by phytoplankton, then subsequently transferred to zooplankton. It then accumulates in fish, and other longer-living species, and ultimately in humans [6,7,8]. Frequent consumption of MeHg contaminated products such as fish, shellfish, and rice, may often lead to chronic toxicity in humans, affecting primarily the kidneys and the central nervous system [8,9,10,11,12,13,14]. A diverse diet will help to avoid the accumulation of Hg in toxic concentrations in humans, but indigenous populations, for example in the Arctic or Amazon, which rely mainly on sea-based food supplies for their everyday nutritional needs, are extremely vulnerable to Hg bioaccumulation [10]. Moreover, due to its mobility in the atmosphere and the globalisation of food supplies, Hg is now found in many food products around the world. In a recent study on the transboundary incorporation of Hg through the food web, the percentage of MeHg, of the total Hg, biomagnified compared to soil, was found to be 13.9% in rice, 38.1% and 48.8% in herbivorous and carnivorous invertebrates respectively, and 92.1% in 10 different species of passerine vertebrates [15]. In addition to MeHg ingestion, human poisoning can occur through skin contact with inorganic Hg^2+^ salts or organomercurials. Acute exposure is commonly the consequence of the occupational inhalation of Hg^0^ vapour, or of the accidental ingestion of inorganic Hg salts [16,17,18,19]. Chronic exposure often results from inhalation of elemental mercury and ingestion of MeHg, for which the minimal risk levels are evaluated at 0.0002 mg m^−3^ and 0.003 mg kg^−1^ day^−1^, respectively [20]. Not only Hg is toxic at such low doses, but Hg exposure is also very subtle and cumulative [13,14,21,22]. The contact exposure pathway strongly relies on the high lipophilicity that both metallic and organic mercury compounds share [18,23]. Hg^0^ crosses cellular membranes, and upon intracellular oxidation binds to sulfhydryl, phosphoryl, carboxyl, amide, or amine groups of enzymes, leading to enzyme inactivation, protein precipitation and generalized corrosive action [16,24,25]. Inorganic mercury exposure represents the least common pathway for human poisoning, potentially leading to gastrointestinal lesions and renal failure [7]. Both organic and inorganic forms of Hg induce apoptosis, disrupt the cellular antioxidant systems, and increase cytokine release. However, there are profound differences in the toxicity mechanisms in each of these processes that are still not entirely understood [24,25].

Mercury occurs naturally in the Earth’s crust from which it is steadily and continuously released into the geosphere through volcanic activity and the constant erosion of rocks. Nevertheless, the development of industrial and technological procedures has dramatically increased Hg mobilization since the industrial revolution. The United Nations’ Environment Programme recently estimated Hg emissions into the air from anthropogenic activities to be as high as 2000–2500 tonnes per year [10]. It is highly disturbing that the anthropogenic emissions of Hg to the air were estimated to have increased by 20% between 2010 and 2015 [10]. Atmospheric Hg emissions in 2008 were estimated as 5207 tonnes, primarily as Hg^0^, from natural sources, especially the oceans, and 2320 tonnes per year from anthropogenic sources, mostly from fossil fuel combustion [26]. However, the contribution from natural sources to atmospheric Hg pollution includes re-emission of both natural and anthropogenic emission-deposition cycles [27]. It is noteworthy that current atmospheric Hg concentrations are 5.5-fold to 7.6-fold higher than pre-anthropogenic values [10,28,29]. The primary contributors to anthropogenic Hg release are artisanal and small-scale gold mining (838 tonnes), cement production (233 tonnes), non-ferrous metal production (228 tonnes), and stationary combustion of coal, gas and oil activities (in total 480 tonnes) [10]. Other anthropogenic sources of Hg include burning of municipal and medical waste including spent batteries, thermometers and electrical products, chloralkali production, vinyl-chloride monomer production, former mining waste disposal sites, agricultural burning and the use of Hg-containing pesticides [10,23]. Special mention should be made of historically contaminated sites, of which there are over 3000 worldwide associated with Hg mining, precious metal processing, non-ferrous metal production, and various polluted industrial sites. These sites represent long-term Hg sources that contribute constantly to an average release of Hg into the environment of 198 tonnes per year: 82 tonnes enter the atmosphere and 116 tonnes are transported by hydrological processes [10,23,30].

The transboundary nature of Hg pollution and its severe toxicity both to humans and to the environment has required strict action to be taken to reduce usage and spread of Hg and to deal with its waste (United Nations Environment Programme, 3rd Conference of the Parties of Minamata Convention on Mercury, Nov. 2019–UNEP/MC/COP.3/INF/28). The objective of the global Minamata Convention treaty, active as of August 2017, is to protect the environment and human health from anthropogenic Hg and other Hg compounds (Minamata Convention on Mercury, 2013, Art. 1). Even though Hg usage has fallen in recent years, abandoned mines and chloralkali plants are still sources of continuous mercury pollution by producing toxic waste, vapour, or organomercurial compounds [31,32]. Technologies such as chemical precipitation, extraction, adsorption, encapsulation, thermal treatment, vitrification, electro-dialysis, are conventional methods of soil and aquatic Hg remediation. However, these technologies are costly and laborious, alter the soil quality, may depend on certain substrate characteristics or pretreatment, and themselves generate hazardous waste that is difficult to recycle, demanding further treatment before disposal [33,34]. Furthermore, engineering technologies are applicable only to small-scale polluted sites, whereas Hg contamination often spreads as far as 50 km from point sources, affecting extensive inhabited and agricultural areas [35,36,37,38,39,40]. Thus, these areas cannot be treated using conventional remediation techniques. In contrast to engineering-based remediation technologies, plants can be used to treat substrates containing contaminants such as Hg without negatively affecting the soil and its biota. Moreover, plant-based strategies to treat contaminated substrates are relatively low in cost and maintenance compared with the engineering procedures. Moreover, plant and microorganism (fungi and bacteria) interactions at the root level (within the rhizosphere) can have synergistic beneficial effects in the remediation of heavy metal contamination in soil. Consequently, increasing interest has been given to creating and implementing combined bio- (the use of microorganisms) and phytoremediation strategies that are environmentally friendly, low in cost and unlikely to generate toxic by-products [41,42,43,44,45,46,47].

The ability to survive on sites with moderate contamination with heavy metals is a striking evolutionary trait shared by many plant taxa. Even more impressive is the acquired hypertolerance mechanism that allows over 700 angiosperm hyperaccumulator species to grow and thrive in extremely heavy metal contaminated substrates [48]. Mercury is particularly known for its potent phytotoxic characteristics. Yet, plant communities develop on sites that are heavily contaminated with Hg, including active or former gold mines around the world [49,50,51,52,53,54,55,56,57]. Many studies have sought to identify the plant species that are best-adapted for growth on these harsh and heavily Hg contaminated soils and on their potential for phytoremediation. Although no hyperaccumulator plant species for Hg phytoremediation have been identified to date; recent research has focused on the role that rhizosphere-inhabiting microorganisms can play in assisting in the phytoremediation of Hg contaminated soil. The presence of Hg in the substrate alters the abundance and diversity of soil microbial communities towards species possessing detoxification mechanisms [58,59,60]. Some microorganisms, including bacteria and fungi, are able to transform Hg species by reducing Hg^2+^, degrading organomercurials, and methylating Hg^2+^ [42,47,61,62]. Additionally, various functional groups present at the cell’s surface can complex and affect Hg speciation and mobility. Moreover, microorganisms can secrete compounds that can be beneficial for plant growth and thus can promote plant survival in Hg contaminated soils. Numerous studies on bacterial Hg-resistance have focused on uncovering intracellular detoxifying mechanisms; however, research on their potential to assist phytoremediation has only recently been addressed. Understanding the interactions between plants native to Hg contaminated soils and their root-associate microorganisms can help to establish efficient and green Hg remediation strategies that could be used for the reclamation of large areas without the need for additional chemical applications or engineering technologies, therefore causing the least ecosystem interference and incurring the lowest costs.

The aim of the present work is to comprehensively review progress with respect to phytoremediation of Hg contaminated soils, using either plant species alone, or plant species and associated microorganisms. Previous reviews on the remediation of Hg contaminated soils have considered engineering-based technologies or chemical applications, sparingly addressing phytoremediation. In recent years, research has focused more on microorganism-assisted strategies, and although important progress has been made, approaches are often disparate. We review current knowledge of phytoremediation and microorganism remediation of Hg-contaminated soils, and identify areas that need further study. We also describe the molecular mechanisms involved in the acquisition, sequestration, and transformation of Hg in plants and microorganisms in Hg contaminated soils.

## 2. Hg Uptake and Detoxification in Plants

Vascular plants have adopted ingenious and diverse strategies to cope with toxic concentrations of heavy metals, including Hg. These comprise avoidance of heavy metal, thus restricting its uptake, stabilization of the metal within the root through binding and sequestration, reduced translocation to aboveground tissues, and sequestration or possible chemical Hg reduction within the leaves. Most terrestrial plants function as excluders with respect to nonessential elements, including Hg. Plants can reduce metal uptake by releasing organic acid exudates such as citrate, malate, and oxalate that chelate the metal ions in the soil solution, thus reducing the bioavailability [63,64]. Plants limit metal uptake by manoeuvring their inner cellular metal tolerance network to set an efficient barrier against the entrance of toxic elements at the root level. To this extent, the root cell wall becomes the binding site of unwanted cations, especially of metals such as Hg that have a high affinity for the negative charges of the cell wall components [65,66,67,68].

The uptake pathways of Hg into root cells are governed by its speciation. The highly lipophilic organic mercurials have a simplified entrance into the symplast network via the root cell plasma membranes. However, the water-soluble ionic forms of mercury (Hg^+^, Hg^2+^) are frequently retained by cell wall components. In the root apoplast, Hg^2+^ can be ligated to oxygen-containing molecules, such as organic acids [69,70,71,72] or to sulphur-rich structural proteins in the cell wall, including extensins and expansins [72]. The uptake of mineral forms of Hg (cinnabar and metacinnabar) has been quantified in the field in plants growing on Hg-contaminated soils, without much being known about the mechanisms employed [72]. Hg trafficking through the root tissues has been mapped in field plants of *Marrubium vulgare* harvested from a highly Hg contaminated site in Spain. Hg binding to thiol rich regions of the root apoplast determined a depletion of available thiol pools starting from the apical regions of the primary and secondary roots (major precipitation sites) towards the epidermis and outer layers of the cortex cells, and all the way up to the xylem vessels [72].

The symplastic uptake of Hg^2+^ is even less investigated and understood. To date, no precise membrane transporter involved in root Hg^2+^ uptake has been identified, though the active process has been inferred in *Lupinus albus* and *Oryza sativa* plants [65,73]. Based on the similarities between Hg and Cd in terms of chemical behaviour, lack of biological purpose and an indirect toxicity effect on plant cells [74], it might be possible that transmembrane metal transport proteins, inadvertently facilitating the influx of Cd, could also be gateways for Hg entrance. In rice, Cd exploits its affinity for essential nutrient plasma membrane transporters, i.e., the natural resistance-associated macrophage protein (Nramp) and iron regulated transporter (IRT) families of proteins [75]. In the order of increased Cd uptake, OsNramp5, primarily a Mn and Fe uptake transporter, is followed by OsNramp1, OsIRT1, and OsIRT2, all defined by a preference for iron [76,77]. Competition for uptake between Mn and Hg was observed in *Lupinus albus* and *Brassica napus* upon addition of Mn to the Hg containing growth solution. The finding implied that Mn and Hg share affinity for the same transporters, but the particularity was only established in *B. napus* [78]. It was proposed that the putative *B. napus* Mn transporter saturated by Hg might be either a homolog of a Mn affinity protein of the Zrt, Irt-like protein (ZIP) family, or a homolog of the AtNramp1, already characterized for its affinity for Mn in *Arabidopsis thaliana*. On the other hand, the Hg influx pattern remained undisturbed in assays where Cu was added as interference, thereby eliminating Cu transporters from the list of Hg uptake possibilities for both plant species [78]. However, competitive interactions between Hg and Cu were inferred in an aquatic system. The uptake of Hg^2+^ in *Elodea nuttallii* shoots from water supplemented with 100 ng HgCl_2_ L^−1^ was inhibited by 97.2% and 80% respectively, upon addition of Cu^+^ or Cu^2+^ [79].

Within the root cells, Hg^2+^ does not maintain a free ionic state. The high affinity that Hg^2+^ has for thiol groups directs it towards cysteine residues. Phytochelatins (PCs) are cysteine-rich peptides enzymatically assembled from units of glutathione (GSH) by phytochelatin synthase (PCS). Ubiquitous across the entire plant kingdom, PCs form covalent bonds with metal ions, including Hg^2+^. Iglesia-Turino et al. [80] have correlated the increased accumulation of Hg in the roots of *Brassica napus* with the tight binding to PCs, especially to PC_2_. In *Brassica chinensis*, the presence of the oxidized Hg-PC_2_, Hg-PC_3_, Hg-PC_4_ and Hg_2_-PC_4_ complexes was understood as the mechanism for sequestering the toxic ion, thus limiting its potential to cause oxidative stress [81]. *Arabidopsis cad1-3* mutant, deficient in PCS and its products, displayed increased sensitivity to Cd, Hg, and As [82]. To complement this phenotype, a genetic construct with the wheat *PCS1* gene, *TaPCS1* was introduced into *cad1-3* plants, expression being targeted either in the root or ectopically. Regardless of where the expression was targeted, Cd, Hg, and As sensitivities were all suppressed in the transgenic plants, with Hg being tolerated in concentrations of up to 10 µM HgCl_2_. Furthermore, the detection of PC complexes in stems and leaves of transgenic lines expressing TaPCS1 to the root alone proved that PC-chelated metals are transported in plants. However, the metal distribution in transgenic lines was only investigated for Cd, for which the translocation rate to the aboveground organs increased linearly with its reduction in roots [82]. Strengthening these findings, the co-expression of two *Escherichia coli* genes, encoding for the enzymes required for GSH synthesis, i.e., glutamate cysteine ligase (GCL) and GSH synthetase (GS), improved growth on mercury-contaminated media in *Arabidopsis thaliana* plants compared to wild-type plants. The content of thiol molecules (GSH, PC_2_ and PC_3_) significantly increased in Hg-treated roots while simultaneously the Hg content of the above ground tissues was three-fold higher in transgenic than in wild-type lines [83].

Free or ligated, toxic or in excess, heavy metals must be sequestered away from the metabolically reactive cell cytosol, and the preferred storage organelle of the plant cell is the vacuole. The tonoplast as a lipid membrane is only semi-permeable, and highly selective for metal or metal-PC complexes due to the abundance of transmembrane transport proteins. To date, two ABC (ATP-Binding Cassette) transporters, AtABCC1 and AtABCC2 with affinity for PC-metal complexes, have been investigated for their potential involvement in Cd and Hg tolerance in *A. thaliana* [84]. The double knock-out mutant *atabcc1 atabcc2* was hypersensitive in the presence of either Cd^2+^ or Hg^2+^, mimicking a phytotoxic behaviour comparable to the one previously reported for the AtPCS1 loss-of-function *cad1-3* mutant. However, proof of the implication that these transporters are involved in metal-PC transfer from the cytosol into the vacuole has only been demonstrated for Cd-PC [84]. Thus, the definitive role of the ABC transporters in Hg-PC transfer is yet to be demonstrated.

Disruption of proteins and induction of reactive oxygen species (ROS) formation are two cytotoxic side effects of Hg binding to the cysteine-rich motifs within proteins. Consequentially, protein misfolding, disruption of structure, replacement of essential co-factor ions and enzymatic activity inhibition occur. These lead to interference in the redox homeostasis of energy-generating cellular processes, photosynthesis and respiration [85,86]. The free radicals released under oxidative stress can eventually override the antioxidant defence system of the cell, and once the GSH pools are depleted and the ROS-enzymes have been overexploited or inhibited, the cycle of damaging oxidation can lead to membrane lipid peroxidation and eventually to cell death [85,86]. Within 24 h of exposure of *Medicago sativa* seedlings to 30 µM Hg, cell redox homeostasis was affected, as shown by the depletion of the GSH pool, increased lipid and protein oxidation and subsequent cell necrosis [87]. *Medicago sativa* seedlings treated with 20 µM Hg displayed a 4-fold increase in malondialdehyde (MDA) content, a consequence of membrane lipid peroxidation. Moreover, increases in the non-protein thiol compounds and in the activity of superoxide dismutase (SOD), guaiacol peroxidase (POD), and ascorbate peroxidase (APX) are indicative of oxidative stress induced by Hg exposure [88]. Furthermore, plants able to tolerate higher concentrations of Hg in their growing media respond by increasing GSH production and the activity of the antioxidant enzymes. Thus, seedlings of *Sesbania drummondii* counteracted the toxicity of 50 mg HgCl_2_ L^−1^ with a 128.8% increase in GSH content, 37.2% in the GSH/GSSG (glutathione disulphide) ratio and 3.86- and 2.62-fold higher activities of SOD and APX respectively [89]. The tendency of plant tolerance attained by enhanced enzymatic and non-enzymatic antioxidant defence systems has likewise been apparent in *Lycopersicon esculentum* [90], *Sesbania grandiflora* [91] and *Cyrtomium macrophyllum* [53]. Moreover, overexpression of genes coding for ROS-scavenging enzymes has also been observed under Hg stress. In wild type *A. thaliana* seedlings, germinated on Murashige Skoog (MS) agar media with 20–40 µM HgCl_2_, the catalase (*CAT3*) was induced by a factor in the range of 2.5–5.0, followed by plastidial Cu/Zn SOD (*CSD2*), with an 1.5–2.5 fold increase [92]. Other components of the redox homeostasis complex system are certainly affected by Hg, but are yet to be identified. Exposure of *Pisum sativum* seedlings to 5 µM HgCl_2_ upregulated the expression of six genes in the roots. Some of these genes are commonly involved in responses to different stresses: *PsPOD* peroxidase, *PsNDA,* a putative NADH dehydrogenase that can adjust the redox balance, *PsAPSR,* a putative adenosine 5-phosphosulphate (APS) reductase involved in sulphur metabolism, isoflavone 2′-hydroxylase (I2′H) involved in the biosynthetic pathway of isoflavonoids and *Ps*SAMPT that catalyses the formation of methylsalicylic acid (MeSA) and thus being involved in plant defence mechanisms against pathogens in roots [93].

There is no robust evidence for Hg^2+^ transport across cellular and vacuolar plant membranes. Transcriptomic and proteomic approaches are required to capture the mechanisms most likely to be involved in Hg metabolism. We suggest that if the experimental design covers gradual concentrations of metal, it would be expected to detect patterns that might facilitate the understanding of Hg transport and detoxification in plants. These mechanisms should be further confirmed by functional complementation of the corresponding knocked-out genes, to establish the grounds for a solid phytoremediation concept.

## 3. Phytoremediation of Hg-Contaminated Soils

Over 3000 Hg polluted sites exist worldwide [94] and often mercury contamination extends up to 50 km from the industrial hot spot [35,36,37,38,39,40]. Moreover, high Hg mobility in the atmosphere facilitates its transport to agricultural lands, leading to it being deposited on formerly uncontaminated areas. Combined with the potential to further redistribute if Hg leaching occurs, the need for remediation of Hg-contaminated soils is clear. In contrast to engineering-based remediation technologies, plants handle contaminants without affecting the topsoil, therefore conserving or even improving its fertility through root exudation [95], and involve low cost and low maintenance. Phytoremediation—the use of plants able to grow and develop in heavily contaminated soils—has therefore been proposed as an alternative, environmental friendly strategy for improving heavy metal-contaminated sites.

An efficient plant remediation system should result in contact with the toxic element and mediation of this interaction to favour its uptake. For most plants, avoiding toxic elements like Hg is their normal behaviour. Therefore, finding plants that do not avoid Hg, or actively seek it out, is an important aspect of phytoremediation that needs to be solved. Plants generally forage for essential nutrients and can even secrete a wide range of chemicals—protons, organic acids, enzymes, phytosiderophores and proteins—to capture them [96,97]. In soil, Hg is largely adsorbed onto organic matter (humic and fulvic acids) or even metallic oxides, and therefore its bioavailability is sometimes low. Decreasing the pH of the soil solution increases mercury mobility. Different chemical chelator-assisted methods have been proposed for increasing the bioavailability of Hg for plants. However, these methods are not detailed herein for the current review focuses on phytoremediation using plants alone or in conjunction with microorganisms.

### 3.1. Phytoextraction

In phytoextraction, plants act as sinks for Hg species, thus the requirements for suitable species to use are having extended root systems, high biomass production and strong cellular Hg-buffering capacity. Hg^2+^ would be uptaken by roots and translocated to shoots where tolerance mechanisms, like chelation with thiol-compounds, vacuolar sequestration, and oxidative stress-alleviating enzymes, are stimulated. At the time of senescence, the Hg-loaded plant material is ready for harvest and soil decontamination is achieved. Yet, the plant biomass should be treated as hazardous and accidental consumption throughout its life cycle would have to be contained otherwise it may contribute to food chain biomagnification [98]. Therefore, this strategy would require proper management and monitoring of the site, and possibly acquiring as well adequate methods for metal recovery from the contaminated biomass.

Some plants have adapted to extreme metal stress by evolving molecular tolerance mechanisms and thus behaving as accumulators or even hyperaccumulators. The definitive trait of an accumulator is its impressive capacity of safely translocating large amounts of metals, symplastically uptaken by root cells, into the aboveground organs [99]. Detoxification is mainly achieved eventually by long-term metal storage inside the vacuoles within the photosynthetically active plant tissue, the leaf mesophyll. Due to the high phytotoxicity of Hg, there have been only a few accumulating plants registered to this date, let alone hyperaccumulators. The accumulation potential of plant species naturally colonizing metalliferous sites is worth exploring both for assessing its *on-site* phytoremediation appropriateness and for elucidating the entryways of toxic metals into the human diet.

In the search for Hg (hyper)accumulator plant species, analysis of native vegetation on Hg-contaminated sites has been undertaken and the results of these studies are summarised in Table 1. The highly desirable phytoremediation potential of Hg accumulating plants has also paved the way for studies aimed at disentangling the underlying mechanisms of plant Hg tolerance. However, a deep understanding of the molecular and structural aspects of Hg accumulation and tolerance in plants is still lacking. Most studies have focused on Hg accumulation by plants, rather than the molecular mechanisms involved (Table 1). In attempting to identify candidate plant species for Hg removal, (hyper)accumulators that are known to tolerate other metals, have been assayed for their capacity to bioaccumulate Hg (Table 1). As the cycle of Hg within the environment is very dynamic, inland waters are often contaminated. Thus, aquatic plant species have been considered for their potential for Hg decontamination in water. *Myriophyllum aquaticum*, *Ludwigina palustris* and *Mentha aquatica* achieved an average Hg removal efficiency of 99.8% when grown in water contaminated with up to 0.5 mg Hg L^−1^ [100]. Investigation of the Hg accumulation potential of *Eichornia crassipes*, *Pistia stratiotes*, *Scirpus tabernaemontani*, and *Colocasia esculenta* in water containing 0.5 or 2 mg Hg L^−1^, attested that all four plant species decreased the water Hg concentration after 30 days to nontoxic levels, based on Microtox analyses [101]. Moreover, *Pistia stratiotes* was able to accumulate quantities of up to 83.2 mg Hg kg^−1^ dw [101].

In an attempt to find more efficient plant species for metal extraction, studies have been conducted on crop plants that already have highly organized cultivation and harvest technologies. Rodriguez et al. [113] investigated the Hg shoot accumulation in barley, white lupine, lentil, chickpea, and wheat grown in substrates with Hg present either naturally bound to soil constituents or in soluble form. When grown in pots with contaminated soil originating from the Almadén region of Spain (32.16 mg kg^−1^ dw), plants were not able to accumulate Hg in proportion to its concentration in the soil. Small concentrations of Hg (0.16−1.13 mg kg^−1^ dw) were detected in the above ground tissues, suggesting that crop plants can only take up the soluble fraction of Hg from the topsoil, leaving the Hg bound to sulphur practically undisturbed.

Accumulation of Hg in plant species used as fodder or for human consumption has been examined because of the high risk they pose to animal and human health. Agricultural plant species cultivated on, or close to Hg contaminated soils, often concentrate alarming levels of Hg within edible tissues, thus posing major concerns for human health. Particular attention has centred on rice, as the staple crop for vast Asian populations. Large areas in China and India are affected by Hg-enriched aerial deposition. Studies from the early 2000s on the redistribution of Hg from aerosols (Hg^2+^) to soil and rice plants in the Hg-polluted area of Guizhou, China, provided useful data on the presence of Hg in rice grains. Surprisingly, in a soil with 33 to 37 mg inorganic Hg kg^−1^, from a sampling point around the Wanshan area, 569 µg kg^−1^ was concentrated in grains, with 145 µg kg^−1^ in the form of MeHg [117]. Following up those findings, the province of Guizhou was estimated to bear a soil Hg concentration ranging from 5.1 to 790 mg kg^−1^. This polluted soil is used to grow other vegetables used for human nutrition [118]. Chinese and baby cabbage, carrot, celery, garlic stem, and *Herba houttuyniae* root can all accumulate over 30 µg Hg kg^−1^ (wet weight), with a maximum accumulation of 128 µg Hg kg^−1^ in the leaves of Chinese cabbage [119]. Vegetal livestock feeding materials, cultivated on Hg-polluted soils are also an important source for Hg accumulation through the food chain. Studies on ryegrass showed accumulation of Hg in shoots significantly exceeding regulations [120]. Further assessments of the toxicity and accumulation of Hg in the edible plant tissues are presented in Table 1. However, few studies on the accumulation of Hg in crops, vegetables, and fodder plants have been included here because the focus of the review is on phytoremediation.

### 3.2. Phytostabilization

Hg phytostabilization operates mainly in the root region, aiming to decrease the metal bioavailability, to immobilize it within the rhizosphere, to increase adsorption onto roots, to decrease uptake and accumulation in roots, and to maintain negligible translocation [98]. This strategy prevents metal dispersal, with almost no accumulation in the aboveground parts of plants, so that specialised disposal of harvested material is unnecessary. However, this strategy does not truly remediate the substrate by reducing the concentration of Hg. While it would decrease Hg bioavailability, it would also require constant monitoring of the species’ Hg content over time.

The use of woody plants, with annually increasing soil-navigating roots, has been proposed as a method for Hg phytostabilization in soils. Fast growing *Salix* spp. have been assessed for their ability to grow under inorganic Hg contamination and for their capacity to stabilize the metal. Different clones of *Salix* spp. were investigated for their growth and Hg accumulation in roots and aboveground tissues. Subsequently, the Hg-tolerant genotype Björn (from *S. viminalis* × *S. schwerinii* E. L. Wolf.) was identified as showing no signs of growth inhibition or water transpiration inhibition when cultivated in solution supplemented with 1 µM Hg (200 µg L^−1^) [121,122]. Moreover, this genotype was able to remove up to 73.9% of the Hg from solution and accumulate very high concentrations of the metal in its tissues (up to 216–274 mg Hg kg^−1^ dw). Based on this Hg tolerance, and on the capacity to retain 80% of the Hg taken up within the roots, the genotype was considered a suitable candidate for phytostabilization of Hg. Investigations on its ability to stabilize Hg within highly contaminated soil (30 mg Hg kg^−1^ dw) were undertaken. It showed high capacity to accumulate Hg in the roots, displaying metal concentrations there of up to 24.90 mg kg^−1^ dw. This exceeded shoot Hg concentrations by a factor of 100 [122]. The profile of the Hg species present in the substrate before cultivation showed that only 0.1% of Hg was water soluble. The majority of Hg was not available for plant uptake as 1.1% of Hg was bound to humic and fulvic acids, while the rest of the Hg ions were retained in complexes. Upon the cultivation of the plants, the only decreases observed in the profile of Hg in soil were for the exchangeable and humic acid-bound fractions, which were accounted for by root uptake. The cation could have been exchanged for the negative charges on the root cell walls. In conclusion, although the quantity of plant accumulated Hg was small (only 0.2% of the total soil Hg), this *Salix* species proved to be a good candidate for the immobilization of the bioavailable form of Hg in contaminated soil [122].

*Salix alba*, another willow species of interest, is frequently used as an ornamental tree around and throughout urban areas. Its ability to accumulate Hg within leaves was assessed in the town of Turda (Romania) [39], where a former chloralkali plant is a constant source of Hg emissions over the town [37]. The average concentration of Hg in the leaves was 340 ng g^−1^, with a maximum value of 4600 ng g^−1^, recorded for a sample collected from the industrial area. Thermal speciation of the Hg species present in plant tissue identified the toxin as MeHg. The implications of this study indicate *Salix alba* leaves as potential sinks for gaseous Hg, with the element being irreversibly contained by conversion into MeHg [39].

*Silene vulgaris*, a perennial with high production that is able to colonize metalliferous soils, is another potential Hg phytostabilizing species. Plants cultivated on two different types of soils (pH 8.55; organic matter 0.63% and pH 7.07; organic matter 0.16%), supplemented with either 0.6 or 5.5 mg Hg kg^−1^ soil, showed no significant decrease in dry biomass compared to plants grown in uncontaminated soil [123]. Plants were evaluated during a complete life cycle of *S. vulgaris*, from February to July and exposed to Hg in soil as HgCl_2_. Roots were the main Hg accumulating organs in both contaminated soils, with significant quantities present in the plants growing in the soil with the higher metal concentration (3.7 and 2.9 mg kg^−1^ dw). Translocation of Hg was significantly lower in the alkaline soil compared to the neutral soil. The high biomass and the low translocation rates registered in a heavily Hg contaminated soil (5.5 mg Hg kg^−1^ soil) make *S. vulgaris* a noteworthy candidate for phytostabilization trials [123].

Plant species growing on highly Hg-contaminated sites are possible candidates for phytoremediation. *Festuca rubra*, *Poa pratensis*, *Armoracia lapathifolia*, *Helianthus tuberosus*, and *Salix viminalis* were cultivated in highly Hg-contaminated soil (261 mg total Hg kg^−1^ dw and 3.1 mg water soluble Hg kg^−1^ dw) collected from the grounds of an operating chemical factory in Poland [124]. Hg did not significantly affect growth parameters of any of the investigated species in comparison to plants grown in uncontaminated garden soil. Roots were the main storage organs for Hg, with the highest concentrations (5.9 mg kg^−1^ dw) found in *S. viminalis*, followed by the two grass species, which had more intricate and deep root networks. *F. rubra* and *P. pratensis* and accumulated 3.0 and 2.7 mg Hg kg^−1^ dw respectively. *H. tuberosus* and *A. lapathifolia* were better excluders with only 1.03 and 0.88 mg Hg kg^−1^ dw in their roots. The cell walls of the outer layers of cortical cells and even central cylinder cells were the main binding sites, although Hg also displayed affinity for parenchyma cell nuclei [124].

*Miscanthus* × *giganteus* (M×G) is a promising tool in phytoremediation because of its high biomass and tolerance to a variety of heavy metals (Cu, Cd, Cr, Pb, Ni and Zn) [125]. Recently, the hybrid was assessed for its capacity to stabilize both Hg and Cd in soils supplemented with low (10 mg Cd kg^−1^ and 2 mg Hg kg^−1^) or high (100 mg Cd kg^−1^ and 20 mg Hg kg^−1^) concentrations of metals [126]. During a 3 years experiment, the biomass yield parameters decreased significantly only in the third year for all plants in all treatments compared to the first growth season. The yield of the plants (tonnes dw ha^−1^) grown in low and highly contaminated soils decreased by 36.3% and 42.7%, respectively compared to that of plants grown in unamended soil. By the second year, the mean accumulation of Hg was statistically significant in the high metal concentration treatment (108.9 µg kg^−1^), while all other treatments and controls had values typically below 30 µg kg^−1^. Generally, the Hg concentration was low in the aboveground tissues, suggesting that Hg is retained in the root epidermis and exodermis. Nevertheless, the biomass removal efficiency of plants grown in the highly Hg contaminated soil was 4.7 µg per pot per year (0.79 g ha^−1^) in the second year [126].

### 3.3. Phytovolatilization

Phytovolatilization implies that plants facilitate the speciation of Hg^2+^ to Hg^0^, concomitant with its transfer from soil into the atmosphere. They should be able to take up Hg^2+^ in roots and distribute it into other tissues that might possess reductive mechanisms. From this point volatile Hg^0^ can diffuse outside the cells or into the xylem, eventually escaping the plant tissues either directly or by stomatal transpiration [98,127]. The plant biomass would not require special attention as Hg species are transiently crossing the tissues without accumulation. However, the Hg species should be bioavailable in the substrate for root uptake, and the release of volatile Hg forms represents a hazard due to potential redeposition, especially in inland areas with low wind speeds that do not promote atmospheric dilution. Catalytic reduction of Hg^2+^ followed by Hg^0^ release into the surroundings occurs naturally in Bacteria, Archaea, and probably in Fungi [128,129,130,131]. Plants might be able to reduce Hg^2+^ by very small imperceptible amounts. Young barley plants can possibly use the available pool of the ascorbate antioxidant system to reduce soluble Hg^2+^ to Hg^0^ and thus emit gaseous Hg from the leaves [132].

*Brassica juncea* has been cultivated in HgCl_2_ spiked Hoagland solution in a controlled, gastight chamber system that made it possible for all Hg to be quantified in all its chemical forms [112] (Table 1). Hg was applied to the growth solution in 0, 0.05, 0.5, 1, 2.5, 5 and 10 mg L^−1^ concentrations. Even though the higher Hg concentrations reduced transpiration rates, the total dry biomass accumulated was similar for all plants. Roots were found to concentrate Hg by 100–270 folds the level in the most contaminated growth solutions, based on dry-weight. Roots also mitigated the volatilization of mercury ions to Hg^0^, a process that was amplified with increasing concentrations of Hg in solution. Root-associated algae or bacteria species were probably responsible for the biochemical volatilization. The high efficiency of Hg removal (95%) from the water was achieved partly by plant root accumulation, but the greatest factor in this experiment was the increased Hg volatilization. On 3 field plots, at the site of an abandoned Tui base mine, New Zealand, Moreno et al. used the Hg-tolerant *B. juncea* to extract Hg from soil and increase the Hg volatilization process by a combined plant-microbe action [133] (Table 1). It is still not clear how plants facilitate the release of Hg from soil to the atmosphere. Photoreductive processes or microbial transformation might convert some fractions of the available Hg^2+^ into Hg^0^ that would be released through the soil particles or through the plant *via* the opened stomata. However, phytovolatilization is primarily achieved by the expression of bacterial enzymatic mechanisms in plants, the transgenic approaches being discussed later within this review.

## 4. Microorganisms-Assisted Hg Phytoremediation

**Figure 1:**
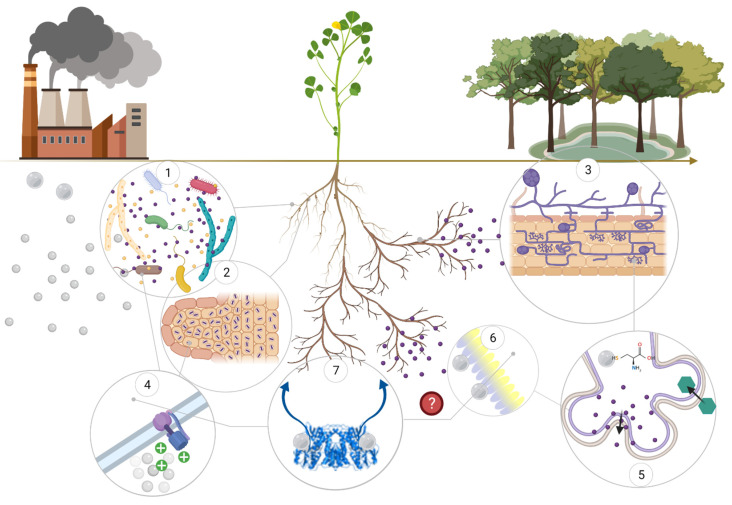
Transition from Hg contaminated sites towards recovered landscapes, exploiting microbial resistance mechanisms in association with plants (**1**) Rhizosphere bacteria and fungi providing minerals (N, P, K, Fe), growth promoting molecules (hormones, enzymes), and Hg-mobilizing molecules (organic acids, amino acids, phenolic compounds) to the root cells; (**2**) Rhizobia during nodulation, providing N to plant root cells, by fixing atmospheric N_2_; (**3**) Extended root architecture with tissues colonized by arbuscular mycorrhizal fungi; (**4**) Hg mobilization by H^+^-ATPase protonation; (**5**) Arbuscular mycorrhizal fungi-plant interface: minerals (purple) are received by the plant cell, while carbohydrates (green) are transferred to fungal cell where Hg is chelated to cysteine-rich proteins; (**6**) Hydrophobin monolayer enveloping fungal cell wall, sequestering Hg ions; (**7**) Reductive volatilization of ionic Hg by bacteria, and putatively fungal cytosolic mercuric reductase. Created with BioRender.com, accessed on 28 February 2021.

Mercury phytoremediation requires plant species to be able to withstand and remove the toxic agent from substrates over extended periods in order to restore the concentration of the metal to an acceptable threshold. Currently, there is still no plant species identified as a Hg hyperaccumulator, increasing the difficulty of Hg soil phytoremediation. Application of chemical compound to soils to either reduce or increase Hg mobility is expensive, unsuitable for large areas and volumes of contaminated soils and potentially damaging for the ecosystem. A promising strategy to overcome these problems involves utilisation of the complex interactions between plants and their associated rhizosphere and/or endophyte microorganisms (bacteria or fungi) (Figure 1).

Microorganisms are ubiquitous within soils and the rhizosphere, and establish intricate and poorly understood interactions with plants. These interactions can be either detrimental or beneficial. Exploiting the latter types of relationships could be an avenue to assist phytoremediation using an environmentally acceptable approach. Moreover, plants often utilise localised microorganisms to aid their survival in inhospitable environments. In doing so, they excrete root exudates required by certain microorganisms that in exchange for the provision of essential organic compounds, provide support for plant growth and protection against competitors and predators.

Before fulfilling a role in assisting phytoremediation of Hg-contaminated soils, microorganisms must first overcome the negative selective pressure exerted by the toxic metal. The common mechanisms used by microorganisms to resist heavy metal toxicity include production of organic acids and polysaccharides that chelate metal ions, biosorption within cell walls and envelopes, intracellular accumulation buffered with cysteine-rich proteins, vacuolar sequestration and chemical transformation [134,135]. Microorganisms are able to modulate Hg speciation in soil by activating specific molecular mechanisms, or by altering the microenvironmental conditions. It has been suggested that sulphur-amino acid-decomposing bacteria within the rhizosphere of *Helianthus tuberosus* and *Armoracia lapathifolia* growing in Hg-contaminated soil immobilize Hg within soil as HgS, by means of sulphur release. Thus, bioavailability of Hg, and consequently root uptake, are reduced [124]. Hg immobilization in soil protects plants against toxic concentrations of Hg, allowing revegetation of Hg-contaminated areas. However, the metal persists in the substrate and continues to pose a potential risk. Hence, to achieve removal of the contaminant via plant uptake, Hg-mobilizing bacterial mechanisms have been considered in the context of assisted Hg phytoextraction.

Mechanisms that enable microorganisms to release Hg^2+^ from complexed forms in soil, increasing its bioavailability for plants, include H^+^-ATPases protonation, organic acids and siderophore secretion, and chemical transformation [134,136]. The ability of bacteria to interfere with Hg cycling, by methylation, demethylation, transport, and reduction of Hg compounds, is encoded under the *mer* operon, the cluster of specific Hg-resistance mechanisms. The emblematic element of *mer* operon is *merA*, encoding for mercuric reductase, a flavin-containing disulfide oxidoreductase, that achieves Hg^2+^ detoxification by reducing it to Hg^0^ that can then diffuse outside the cell [128]. In addition to MerA, which enables narrow-spectrum Hg resistance, the enzyme organomercurial lyase, encoded by the *merB* gene is present in broad-spectrum Hg-resistance. The enzyme cleaves the C-Hg bond in organomercurial species by a protonolytic attack, enabling further Hg^2+^ reduction to be conducted by mercuric reductase [128]. Extensively, other components of the *mer* operon are *merT*, *merP*, *merC*, encoding for transport proteins, and *merR* and *merD*, which encode for regulatory elements [128]. The increased mobility and bioavailability of Hg in soil, mediated by bacterial processes, would facilitate plant uptake, leading to improved phytoextraction, while Hg^2+^ volatilization would augment the overall process.

The fundamental trait required in phytoextraction is increased plant biomass, with a significant amount of Hg accumulated within tissues without impairing plant growth. Mercury adversely affects seedling and root development in non-tolerant plants, photosynthesis efficiency and metabolic homeostasis, leading to a reduction in plant mass. However, even Hg-tolerant plants that might be useful in phytoremediation exhibit similar responses when exposed to toxic Hg concentrations in soil. Some of the plant-associated microorganisms, including bacteria and fungi, can promote plant growth and thus act as important contributors to plant adaptation to environmental stress caused by heavy metal contaminants. These microorganisms, collectively named plant growth promoting microorganisms (PGPMs), can assist plant growth and reduce metal toxicity either by aiding the acquisition of essential mineral nutrients or by restraining the acquisition of nonessential or toxic minerals or substances. Plant partnerships with Hg-tolerant PGPMs can enhance germination, survival and growth in Hg-contaminated sites. Nevertheless, some microorganisms are able to induce plant defence mechanisms against pathogens and thus, indirectly increase plant fitness and survival [137,138,139].

Another desirable trait in phytoremediation, that is significant especially for large-scale contaminated areas, is the ease of plant establishment. Sowing seeds should be a suitable approach for phytoremediation of open fields, rather than using plantlets, which are costly to grow, time-consuming to plant, and may have a low planting success rate. Accompanying appropriate microbial inoculums could provide crucial support for plants at all developmental stages, from seed to maturity, against the abiotic stress of the toxic element.

The success of microbe-assisted phytoremediation depends on two main effects that microorganisms can exert within the rhizosphere. Firstly, the type and quantity of molecules secreted by PGPMs must confer a survival advantage to relevant plant species under stressful conditions. Secondly, microorganisms should help to increase metal bioavailability, solubility and accumulation in plants, when decontamination rather than stabilization is the aim.

### 4.1. Bacteria-Assisted Hg Phytoremediation

Plant growth-promoting bacteria (PGPB) comprise a heterogeneous group of prokaryotes inhabiting diverse ecological niches, including rhizosphere free-living (rhizobacteria), root nodule inhabiting (rhizobia) or plant interior tissue-inhabiting (endophytes) species. PGPB are able to stimulate plant growth, acting as biofertilizers, phytostimulators, bioalleviators, biopesticides, and biomodifiers [138,140]. Exchanges between PGPB and associated plant species facilitate survival and growth of both partners under toxic metal stress. Bacterial mechanisms that promote plant growth and protection against phytopathogens are numerous, and often not thoroughly characterized. In general, PGPB provide valuable nutrients (e.g., fixed N, Fe, P), signals for induction of systemic resistance, e.g., volatile organic compounds, hormones (e.g.*,* abscisic acid, ethylene, jasmonate, cytokinins, gibberellins, indole-3-acetic acid (IAA)), enzymes (e.g., 1-aminocyclopropane-1-carboxylate (ACC) deaminase, chitinases, cellulases, proteases, lipases), antibiotics or siderophores [138,140,141].

#### 4.1.1. Rhizobial Associations

Research on the Hg-plants-bacteria triad has mainly been focused on assessing the potential of Hg removal from soil, upon association between plants that are often indigenous to contaminated sites, and symbiotic bacteria. Examination of the potential use of legume-rhizobia associations that are native to contaminated sites, is a pragmatic and practical strategy for Hg phytoremediation. Legume-rhizobia compatibilities are already settled and, as rhizobia sustain plant growth, they increase plant ability to cope with Hg stress. In the Almadén mining district in Spain, which is contaminated mainly with Hg, native vegetation, such as *Trifolium* spp. and *Medicago* spp., have established specific associations with beneficial *Rhizobium leguminosarum* bv. *trifolii*, and *Sinorhizobium* (*Ensifer*) *medicae* respectively [142]. Distinct bacterial isolates showed wide phenotypic diversity in terms of Hg, pH and salt tolerance, and phosphate solubilization, but no siderophore production [142]. These findings highlight the taxonomic selectivity that plants employ when establishing microbial associations, but the lack of perception of Hg-tolerant strains. *Lupinus albus* L. plants were able to nodulate, to accumulate about 370 mg Hg kg^−1^ in roots, and about 360 mg Hg kg^−1^ in nodules, and to maintain constant levels of photosynthetic pigments, when inoculated with Hg-tolerant *Bradyrhizobium canariense* L-7AH strain, but not with the Hg-sensitive strain L-3, both isolated from the Almadén mining site soil, in substrate containing up to 102 mg Hg kg^−1^ [41]. However, the resistance mechanisms of these isolates are not understood, as attempts to detect the *mer* operon elements were unsuccessful [143].

#### 4.1.2. Non-Rhizobial Associations

The possibility of non-rhizobial, but Hg-resistant isolates being used to aid in Hg removal has been investigated in recent years. A Hg-reducing bacterium, *Photobacterium* sp. strain MELD1, a symbiont from the rhizosphere of *Phragmites australis* plants, that is native to Hg- and other heavy metal-contaminated sites, showed promising phytoextraction potential. *Vigna unguiculata* ssp. *sesquipedalis* inoculated with *Photobacterium* sp. and cultivated in contaminated soil (on average 27 mg Hg kg^−1^), showed increased root length (11%), seed number (33%), leaf number (54%), Hg uptake in roots (25%), and decreased Hg content in aerial organs (55%) in comparison to the uninoculated control [42]. Two heavy metal-resistant rhizobacteria, *Brevundimonas diminuta* SF-S1-5 and *Alcaligenes faecalis* SF-S1-60, applied individually, assisted *Scirpus mucronatus* growth in sand containing a mixture of 100 mg Pb kg^−1^ and 1 mg Hg kg^−1^ over 42 days. The presence of bacteria enhanced phytoaccumulation relative to the uninoculated plants, by up to 650 and 320 mg Pb kg^−1^ and up to 7.5 and 2.4 mg Hg kg^−1^ in roots and shoots respectively [144]. Growth and Hg^2+^ uptake capacities were improved in *Salvinia natans* and *Lemna minor* grown in aqueous solution containing 0.3 mg Hg(NO_3_)_2_ L^−1^, by the presence of three strains of epiphytic bacteria [145]. Hg-resistant *Enterobacter ludwigii* and *Klebsiella pneumoniae*, isolated from *Alternanthera sessilis* and *Cyperus esculentus*, growing on a Hg-contaminated site individually stimulated growth, lowered proline, malondialdehyde content, and electron leakage in commercial *Triticum aestivum* seedlings under 75 μM HgCl_2_ in hydroponics [146]. A consortium of 5 Hg/As-resistant bacteria isolated from a Hg/As highly contaminated soil, and selected on the basis of their ability to produce IAA, ammonia, exopolysaccharide, biofilm, or to fix N_2_, was tested in association with thiosulfate, a fertilizer acting as a metal mobilizing agent, for its potential to improve phytoextraction [147]. The inoculum acted synergistically in combination with thiosulfate, enhancing Hg phytoaccumulation by 35.8% in *Lupinus albus* and 44.7% in *Brassica juncea* [147].

Multi-metal resistant bacteria, endophytes of *Aeschynomene fluminensis* and *Polygonum acuminatum,* sampled from moderately Hg contaminated soils were investigated for their capacity to assist crop plant species growth under Hg^2+^ stress. The presence of endophytic bacteria increased *Zea mays* biomass relative to plants grown in the absence of Hg^2+^, when grown in soil supplemented with 80 mg Hg^2+^ kg^−1^, and reduced the metal concentration in the substrate, by up to 63.19%, mostly due to volatilization [47]. Root and aerial biomass of inoculated *Zea mays* was 74% and 26% higher respectively, with 20-fold greater Hg concentrations in roots than in shoots [47]. Inoculation of *Zea mays* seeds with different rhizosphere bacterial isolates from the above-mentioned plants increased growth in diameter and length of root and shoot by up to 47% and dry mass by up to 51% compared to uninoculated plants, when grown in soil containing 40 mg Hg^2+^ kg^−1^ dw. Furthermore, the association promoted uptake of up to 923 mg Hg kg^−1^ in roots of maize plants, but diminished translocation efficiency to the leaves [46]. These results clearly demonstrate the beneficial effects of certain soil, rhizosphere and endophytic bacteria on Hg phytoremediation. Although PGP abilities were not consistently assayed across these studies, IAA, NH_3_ production and N_2_ fixation were often detected for the investigated bacterial isolates. With respect to Hg resistance, all isolates were able to grow in the presence of various Hg concentrations, and some even reduced Hg concentration in the substrate independently of the presence of plants. However, although it was not examined in all instances, the *merA* gene was not always detected. Importantly, in addition to sustaining and improving plant growth under toxic conditions, rhizosphere bacteria strongly promote an essential aspect of phytoremediation, namely Hg uptake by roots. However, the mechanisms by which bacteria promote root Hg uptake are still poorly understood.

#### 4.1.3. Bacterial Siderophores, IAA, NH_3_

Siderophores are small iron-chelating compounds secreted by both plants and microorganisms that increase Fe solubility in soil and uptake. There is also evidence that siderophores increase the resistance/tolerance of plants to heavy metal contamination. Thus, the naturally-occurring Ni-resistant microbial community in *Thlaspi goesingense* at a Ni-contaminated site was able to produce siderophores [148]. The Cd-hyperaccumulator *Solanum nigrum* benefited from association with its endophyte, *Pseudomonas* sp. Lk9, which synthesizes siderophores, biosurfactants, and organic acids, increasing Cd, Zn, and Cu availability, thus enhancing phytoextraction compared to uninoculated control plants [149]. Bacterial siderophores have been proposed as chelation agents in heavy metal remediation [150], but their interaction with Hg has scarcely been investigated and results are inconsistent. Induced siderophore synthesis in the presence of Al^3+^, Cd^2+^, Cu^2+^, Ni^2+^, was demonstrated for three metal-resistant *Streptomyces* spp., and the binding of Cd and Ni to these molecules was confirmed [151]. A stimulatory effect on siderophore production in *Pseudomonas aeruginosa* strain PAO1 was documented for Cd, but not for Zn or Hg [152]. However, a *Pseudomonas aeruginosa* siderophore—pyoverdine–was shown to chelate Hg^2+^ extracellularly, forming a complex that is internalized *via* a cell surface localized transporter [153]. Although the presence of Hg does not induce siderophore production, the presence of the Hg-siderophore inhibits uptake of the Fe-siderophores complex [153].

It is possible that in the rhizosphere, Hg^2+^, mobilized by bacterial secreted biosurfactants, organic acid, or by protonation, would chelate to siderophores and eventually cross the plants’ cells, leading to phytoaccumulation. It has been hypothesized that PGPB might influence root heavy metal uptake by regulating major metal transporter gene families in plants [154], but evidence is lacking in relation to Hg transport. However, siderophores do not always promote heavy metal uptake in plants, therefore the contrasting effects of siderophore-producing bacteria on heavy metal uptake in plants suggest a plant-dependent metal uptake mechanism [155]. Moreover, not all PGPB are able to produce siderophores. The role of these compounds in relation to Hg chelation and transfer to root cells is elusive. Nevertheless, they might hold a key for enhancing Hg root uptake if proven to outcompete Fe.

**Figure 2:**
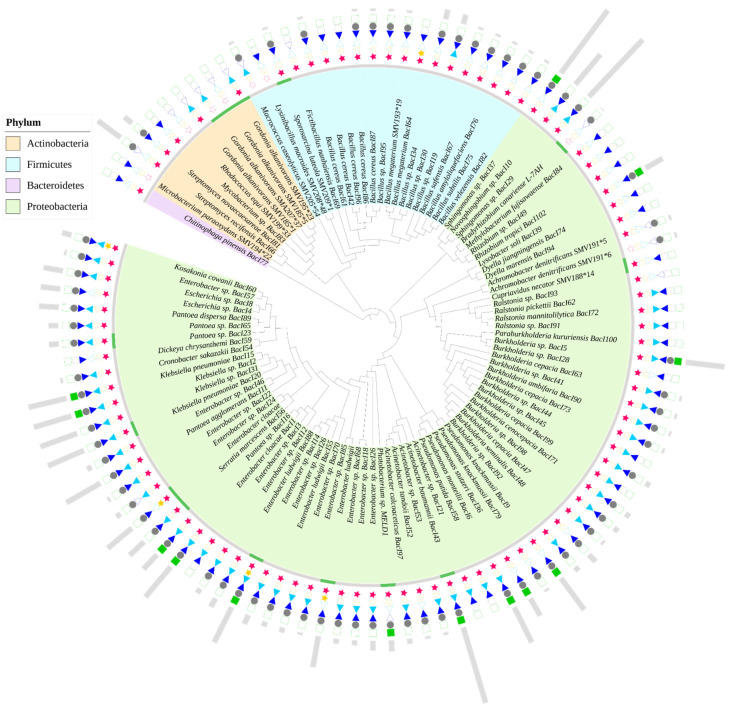
Neighbor-Joining tree, based on Poisson model, of bacterial isolates investigated for their Hg phytoremediation enhancement potential. The full/empty symbols represent the presence/absence of traits relevant to Hg phytoremediation: red ★—IAA production, yellow ★—siderophore production, light blue ◀—N_2_ fixation, dark blue ▶—NH_3_ production, grey ●—Hg resistance, green ▄—merA. The green bands in the grey ring represent the isolates that were tested for their improvement of Hg phytoremediation, while the external grey bars represent the minimum inhibitory concentration value for Hg. The tree was generated in MEGA X [157], using 16S DNA sequences collected from [41,42,47,56,147], and processed in iTOL [158].

A detailed phylogenetic tree is presented (Figure 2) for the identified bacterial isolates that have been screened and studied to date, in relationship to their potential for improving Hg phytoremediation and their PGP abilities. The source of isolates included in Figure 2 varies both in terms of the substrate from which they were derived (bulk soil, rhizosphere soil, root nodules, root endophytes), and in the levels of Hg contamination [41,42,47,56,147]. The phylogenetic tree therefore captures bacterial species that are likely to be recruited by plants in Hg-contaminated soils. It also displays the incidence in the bacterial isolates of the Hg resistance and PGP traits that are relevant for Hg-phytoremediation (Figure 2). The analysis reveals that Proteobacteria phylum has the highest proportion (72% of total isolates) and diversity (23 genera), followed by Firmicutes (18% of total isolates with only 5 genera). Proteobacteria is usually the most abundant phylum in uncontaminated soils where the rhizosphere bacterial communities are often represented by Proteobacteria > Actinobacteria > Bacteroidetes > Firmicutes [156]. However, in Hg-contaminated soil, the rhizosphere bacterial community structure exhibits greater species richness and diversity, with the phylum Firmicutes most abundant, and often displaying siderophores, IAA secretion, ammonia production and the *mer* operon [46].

Further comprehensive investigations of the rhizosphere and endophyte microbiomes and of their PGP abilities, are needed to shed light on the distinct roles played by each taxonomic group in assisting Hg phytoremediation by candidate plant species. The need for research on PGP traits was highlighted previously, as host plants might select for beneficial traits scattered across different taxonomic groups, rather than for specific taxonomic groups [156]. Moreover, Rhizobiales, such as *Bradyrhizobium* spp., *Rhizobium* spp., and Pseudomonadales, such as *Acinetobacter* spp., *Pseudomonas* spp., that are included within the tree, represent a universal core microbiome, probably due to their conserved adaptation to the plant environment [156].

Of the bacterial isolates within the tree, Hg resistance was widespread among Proteobacteria, with minimum inhibitory concentration values spiking above 500 μM for about 15% of the assayed isolates. None of the isolates from other phyla (Firmicutes, Bacteroidetes and Actinobacteria) was able to withstand such an elevated concentration. Of 29% of the strains that were screened for *merA* presence, 45% were positive, and their tolerance level seems to be correlated with gene presence. It is likely that *merA* is widespread among bacteria from Hg-contaminated sites, especially for variants encoded by plasmids that enable sharing of this advantageous trait.

In terms of PGP abilities, IAA and NH_3_ production are frequent in Proteobacteria and Firmicutes, while N_2_ fixation and siderophore production are the least commonly detected beneficial traits. Indeed, IAA production is common in soil bacteria. About 80% of rhizosphere bacteria synthesize this hormone, usually from tryptophane that is released in root exudates [159,160]. Bacterial synthesis of IAA is associated with root proliferation when the released IAA quantity is optimum for the associated plant species. However, the positive effects of bacteria-produced IAA on plant phenotype are seen only in conjunction with other PGP mechanisms [161,162,163,164], but bacteria-producing IAA might confer a significant advantage to plants in Hg phytoremediation field situations because it stimulates rooting. NH_3_ production is often associated with N_2_-fixing bacteria. It may also be produced by ACC deaminase acting on ACC. Therefore, the spectrum of microorganisms able to provide plants with an accessible N source extends beyond Rhizobia and other non-symbiotic N_2_-fixing bacteria. Although siderophore-producing ability was screened for in 86% of the isolates considered, it was a rare trait in bacteria inhabiting plants in Hg-contaminated soils. Conversely, siderophore production was frequently associated with bacteria from heavy metal-contaminated substrates [155]. As plants secrete phytosiderophores, or recruit siderophore-producing bacteria under Fe-limiting conditions, it is possible that for the source of selected isolates this was not the case.

The bacterial candidates proposed for in situ Hg assisted-phytoremediation often originate from sites in need of decontamination, in preference to using non-indigenous, potentially invasive species. However, a recent study has shown that PGPB inoculation of *Brassica juncea* seedlings grown in Cd, Cu, Pb, Zn, As, Mo-contaminated soil can be beneficial. The non-indigenous inoculum does not significantly alter the composition of the resident bacterial communities over time, but it has also been suggested that it may integrate into these complex communities and intensify their interaction, creating a more efficient community that synergistically resists heavy metals [165]. Nevertheless, plant and bacteria interactions are complex, and the use of non-indigenous species should be carefully considered and assessed before designing large-scale field trials.

### 4.2. Fungi

Mycorrhizal fungi may live inside the plant root cortex, on the root surface, or around the epidermal root cells, and supply plants, by virtue of an extended hyphal network, with phosphates, nitrates or organically bound nutrients that are otherwise unavailable, including exchange of carbohydrates [166,167,168]. Moreover, when associations are established, they can facilitate resources and chemical signal transfer between different plant individuals through the hyphal network [166].

Similarly to studies of bacteria, studies on fungal-assisted Hg phytoremediation have focused mostly on symbiotic partners, i.e., arbuscular mycorrhizal fungi (AMF). Interesting findings were revealed involving association of an AMF, *Glomus mosseae*, with *Zea mays* in artificially contaminated soil, with 1, 2, 4 mg Hg^2+^ kg^−1^: the fungus promoted Hg evaporation and Hg soil sorption, thus reducing its bioavailability, and subsequently root uptake compared to soils free of inoculum [169]. Similarly, soil inoculation with a commercial arbuscular mycorrhizal formula, containing *Rhisophagus irregularis* and *Funneliformis mosseae* propagules contributed to Hg imobilizatin in soil in combination with humic acid. The AMF formula increased plant growth and P uptake and reduced Hg root uptake and translocation in *Lactuca sativa*, under 10 mg Hg kg^−1^ dw treatment [170]. Commercial arbuscular mycorrhizal formula, represented by *Glomus, Entrophospora* and *Scutellospora* genera, improved *L. sativa* seedlings development and promoted root elongation, even at 100 mg Hg kg^−1^ dw contaminated substrate, relative to uninoculated seedlings [171]. Commercial arbuscular mycorrhizal formulas associated with *Lolium perenne*, in soil containing 1 mg Hg^2+^ kg^−1^ dw, resulted in higher root uptake (0.49 vs. 0.12 mg Hg kg^−1^ dw), lower translocation (0.28 vs. 0.75 mg Hg kg^−1^ dw), and lower overall metal removal from substrate (65.8%), compared to non-inoculated control (75.6%) [172]. Although these AMF inocula, which are non-indigenous to contaminated sites, stimulate plant growth and protect against Hg toxicity by reducing its bioavailability, metal removal is not achieved. *Glomus* sp., isolated from a Hg-polluted site, stimulated Hg uptake in *Zea mays* cultivated in soil artificially contaminated with 50 mg Hg^2+^ kg^−1^ dw [44]. The concentration of Hg in these maize plants was up to 439 mg kg^−1^, more than twice as high as with a commercial arbuscular mycorrhizal formula and in uninoculated plants. It was hypothesized that fungal metallothioneins (MTs) or PCs could have bound the metal and transferred it to the roots, as the tetrathiol Hg complexation detected was correlated with the fungus samples [44]. In contrast, AMF from a Hg-contaminated site was less effective than a commercial inoculum in terms of promoting plant biomass and photosynthetic pigment content when associated with *Zea mays*, in soil containing 50 mg Hg^2+^ kg^−1^ dw [173]. Its presence increased the metal’s mobility both from soil to roots and within the plant, and impaired mineral uptake [173]. It was concluded that the inoculum native to the contaminated site might not have been compatible with the plant, and that although it was adapted to Hg-contaminated soil, its negative effects on plant growth exceeded any beneficial effects it had on Hg uptake. These studies suggest that mycorrhizal fungi need to have adaptations to Hg toxicity to promote phytoextraction, and compatibility with the host plant.

Colonization of potential Hg-hyperaccumulator plants with AMF can be a strategy for Hg phytoremediation. *Erato polymnioides* individuals, sampled from gold mine soils polluted with Hg, had the highest capacity for Hg accumulation in roots among the plant species present at the site (Table 1). This could have been due to the high percentage of AMF colonization. Although root-to-leaf Hg translocation was relatively low, the concentration of Hg in the aboveground organs was still significantly higher than in the other assessed plant species (Table 1) [103]. Compared to uninoculated plants, *Chrysopogon zizanioides* cultivated individually with 2 commercial AMF showed improved growth and increased Hg uptake in 4 weeks, but only in the most contaminated soil (6 mg Hg kg^−1^ dw) [174]. Four highly Hg-tolerant fungal root endophytes, *Aspergillus* sp., *Curvularia geniculata* P1, Lindgomycetaceae P87, and *Westerdykella* sp. P71, applied individually or in combination, increased *Aeschynomene fluminensis* and *Zea mays* root dry weight by 43% to 196%, shoot dry mass by 40 to 59%, and chlorophyll index and Hg^2+^ accumulation, by 40% and 34% respectively. This was mostly due to decreased Hg^2+^ translocation compared to uninoculated plants [175]. From these studies, it can be assumed that the effects of AMF in Hg phytoextraction involve increasing plant growth and Hg root uptake, and lowering Hg translocation to the shoot. But these effects only occur if the association between the plant and fungus is compatible. How fungi facilitate the transfer of metal from soil into plant tissues is still unclear. Because metal analysis of plant organs can only be done in the presence of the fungal colonizer, it can not be determined whether Hg has been transferred from the fungus into the plant’s cells, or whether it remains bound to the surface of fungal cells, or chelated intracellularly. Nevertheless, it is possible that the fungal network developed within plant tissues, in AMF associations act as a Hg-screen. This would explain the higher Hg concentrations detected in roots and the tendency for reduced Hg translocation in such interactions.

Fungi can take up Hg^2+^ and reduce it to Hg^0^, as the intracellular presence of metallic Hg was reported to occur in aquatic environments contaminated with HgCl_2_ [61]. Fungal MTs and PCs–low molecular weight cysteine-rich proteins involved in heavy metal resistance [176,177,178,179]—might chelate cytosolic Hg^2+^ and sequester it within vacuoles or even relocate it to the plant’s root cells. Hydrophobins are proteins of low molecular mass that are unique to fungi, that are rich in cysteine residues, like MTs and PCs, and able to assemble into amphipathic monolayers, with high surfactant activity [180]. These might be involved in Hg sequestration at the interface with the environment. Puglisi et al. [181] indicated a possible role of these molecules in Hg resistance, as the transcript level of two hydrophobins was clearly upregulated by the Hg treatment.

Thus, the repertoire of fungal Hg-resistance mechanisms is diverse and robust, and AMF candidates have already proved beneficial for Hg phytoremediation. However, understanding of the molecular mechanisms involved in Hg transfer at the plant-fungus interface is still scarce. Moreover, studies on representatives other than AMF in relation to Hg phytoremediation are lacking.

## 5. Are Transgenic Plants Ready for Hg Phytoremediation?

**Figure 3:**
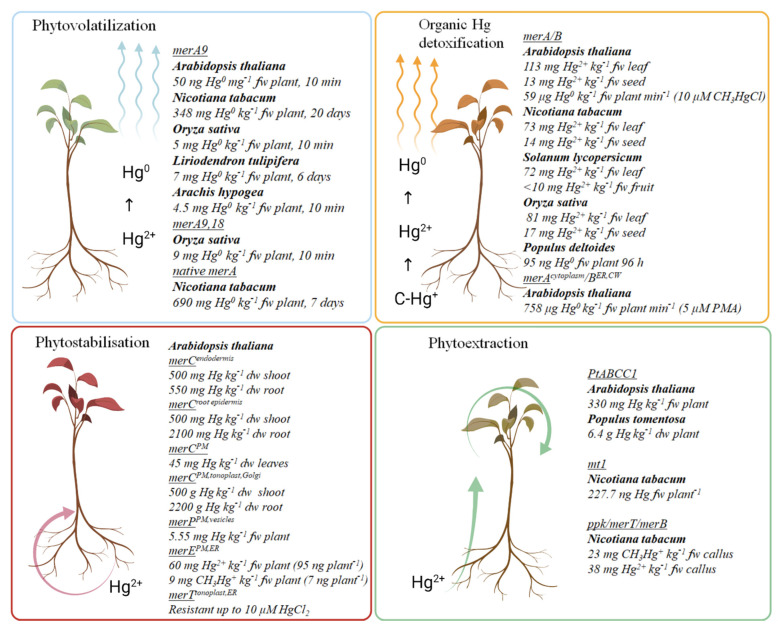
Schematic representation of transgenic plants expressing genes involved in Hg detoxification reported throughout the literature. The plant species are enlisted under each gene they are transgenic for, and the maximum concentrations or quantities detected in tissues or volatilized are adjacent. The genes were clustered by their phytoremediation outcome: phytovolatilization, which enables the reduction of inorganic Hg^2+^ to volatile Hg^0^; organic Hg detoxification, initiated by dealkylation of organic Hg compounds by MerB, resulting Hg^2+^, and followed by reduction to Hg^0^, if in conjunction with MerA; phytostabilization, which speeds up mercury uptake and its immobilization within root tissues; phytoextraction, which enhances tolerance by augmented Hg-buffering systems; ER—endoplasmic reticulum, CW—cell wall, PM—plasma membrane, dw—dry weight, fw—fresh weight, PMA—phenylmercury acetate. Created with BioRender.com, accessed on 28 February 2021.

Genetic engineering may enable traits promoting phytoremediation to be introduced into suitable recipient plant species. To date, most attempts to achieve this have aimed to increase heavy metal accumulation capacity and speed, and tissue specificity for metal uptake, through expression in recipient plants of bacterial genes involved in Hg detoxification (Figure 3). Most transgenic plants for Hg phytoremediation are based on the bacterial *mer* operon, the most effective and widely-studied Hg resistance mechanism. First attempts were based on *Arabidopsis thaliana* and *Nicotiana tabacum*. Encouraging results led to trials being extended to crop species and aquatic species, addressing the problematic bacterial Hg methylation. However, regulations associated with the hazardous potential of transgenic plants have so far prevented field investigations being carried out.

The bacterial native *merA* gene encoding for mercuric reductase, isolated from an environmental *E. coli* plasmid, was successfully integrated into the genome of *N. tabacum* and translated to active MerA [182]. However, the native *merA* had firstly to be engineered for satisfactory expression of the functional enzyme in plants. Hence, a modified *merApe9* gene was constructed by replacing 9% of the coding region with nucleotide combinations and codons more common in plants [183]. The transgenic plants obtained were able to develop all life stages in medium containing HgCl_2_ up to 140 μM, and the MerApe9 mRNA level was correlated with higher rates of Hg volatilization and resistance. Following this, further engineering of phytovolatilization capacity was carried out. Transgenic lines of *Liriodendron tulipifera* [184]*, Nicotiana tabacum* [182,185,186]*, Arachis hypogea* [187]*, Populus deltoides* [188]*,* and *Oryza sativa* [189] showed increased Hg resistance and volatilization capacity relative to wild type. When assessed, Hg accumulation was lower in *N. tabacum* and *O. sativa* transgenic lines than in wild type plants [186,189], while Hg volatilization in the *N. tabacum* transgenic lines was mostly carried out in the root system [182,185,186]. This suggests that once Hg is taken up by the roots it is readily volatilized before moving into aboveground tissues [182,185,186]. Grafting experiments of *N. tabacum* wild type stem on *merA* roots, showed good mobilization of Hg in roots, followed by stabilization in shoots. It was concluded that this could have been due to accelerated cycling between Hg^2+^ and Hg^0^ in *merA* tissues that delivered Hg^0^ to the untransformed stems where it was reconverted to Hg^2+^ and subsequently bound to intracellular chelators [186]. Moreover, among the transgenic lines obtained, which presented different rates of Hg translocation and accumulation in leaves, sterile lines were identified. These transgenic lines were proposed for safe field application by asexual propagation, since the dispersal of pollen or seeds could not occur [189]. However, phytovolatilization is restricted to the remediation of inorganic Hg species.

The detoxification mechanism of organic mercury compounds to less harmful Hg^2+^ and then to Hg^0^ species—referred to as bacterial broad-spectrum resistance—was sequentially transferred to plants. *Arabidopsis thaliana*, ecotype RLD, transgenic lines expressing *merB*, from plasmid R831b encoding for organomercurial lyase, and *merA/B* were generated. Expression of *merB* in *A. thaliana* confirmed the resistance phenotype to organic Hg [190]. Subsequently, the *merA/B* lines confirmed that both genes are required for detoxification of organic Hg to Hg^0^ [191]. Subsequently, MerB-targeted expression in the endoplasmic reticulum for secretion to the cell wall, in addition to the cytoplasmic MerA, increased the efficiency of processing organic Hg [192]. The mechanism was also transferred to *Populus deltoides*, a more suitable candidate for field remediation, which is able to develop large biomass, and a deep root system, making it valuable for long-term usage. *Populus deltoides* transgenic for *merA/B* was superior to wild type and to single gene transgenic lines in terms of resistance and detoxification of organic mercury compounds [193]. The *merA/B* constructs were integrated into the *N. tabacum* chloroplast genome, to protect this organelle, which is very sensitive to Hg toxicity, and to ensure the production of large numbers of transgenes per cell [194]. Organic and inorganic Hg accumulation increased up to 3-fold in roots and 100-fold in shoots relative to untransformed plants, and the transgenic lines attained high tolerance by rapid volatilization. However, the correlation between the *merA* and *merB* transcript levels, and the volatilization capacity were not assayed. Recently, *Solanum lycopersicum* and *Oryza sativa merA/B* transgenic lines were proposed as safe crops for remediation of Hg contaminated soil, since they were able to grow at organic and inorganic Hg concentrations toxic to the wild type, and to remove Hg from soil without accumulating it in their leaves, grains and fruits [195]. Thus, transfer of the bacterial broad resistance mechanism to plants can lead to *merA/B* transgenic lines capable of decomposing organic Hg and further volatilizing it to Hg^0^. However, although presumably organic mercury readily crosses cell membranes due to its lipophilicity as a consequence of a gradient concentration, there was no control over the first step required prior to Hg detoxification, namely root uptake of Hg in either organic or inorganic form.

In addition to the Hg detoxifying enzymes, the *mer* operon may contain genes expressing *merC*, *merT*, mer*P* transporters, that internalize Hg^2+^, and *merE* that internalizes both MeHg and Hg^2+^ in bacteria. Some studies have attempted to increase the speed of phytoextraction by expressing bacterial specific Hg transporters. Increased inorganic or organic Hg accumulation was reported for *A. thaliana* transgenic lines for *merC*, *merP*, *merT* and *merE*. The transporters were frequently expressed within the plasma membrane, Golgi apparatus, and vacuolar membrane, providing reduced toxicity compared to the wild type. That may be due to vacuolar compartmentalization of the contaminant and chelation of thiol-rich compounds that would decrease ROS production [196,197,198,199,200]. Expression of *merC* was achieved in *A. thaliana* in both root epidermis, under the pEpi promoter and endodermis, under SCARECROW promoter, exhibiting higher Hg accumulation in shoots relative to wild type, and comparable to constitutive overexpressing lines, indicating that tissue-specific expression of transport proteins is sufficient to achieve enhanced Hg translocation [201,202]. *N. tabacum* was sequentially engineered with (1) enhanced cellular Hg sequestration *via* bacterial *ppk*, encoding for polyphosphate kinase, the key enzyme in the synthesis of the negative polyP polymer capable of binding metal ions, with (2) *merT* for accelerated inorganic Hg uptake, and (3) with *merB* to extend the spectrum resistance [203,204,205,206]. This conceptualization came as a solution to the alarming phenomenon of Hg volatilization *via merA* transgenic lines. It effectively provided accelerated uptake of inorganic Hg, detoxification of organic Hg, and enhanced accumulation in plant material from which Hg could be recovered and recycled [206]. Similarly, *mt1*, a mouse MT, was integrated into the *N. tabacum* chloroplast genome, generating transgenic lines able to accumulate up to 4.5-fold higher concentrations of inorganic Hg in all tissues relative to untransformed plants [207]. In addition, overexpression of *PtABCC1*, a tonoplast-localized ATP-binding cassette Hg-chelate transporter from *Populus trichocarpa*, proved effective in *A. thaliana* and *Populus tomentosa* in promoting growth under 20 μM HgCl_2_ and in increasing Hg accumulation both in roots and aboveground tissues compared to wild type plants [208]. The strategy of improving the plant tissue tolerance to elevated Hg levels by constitutive overexpression of mechanisms that buffer Hg toxicity, gives the possibility of establishing phytoremediation communities in heavily contaminated sites, especially for high biomass producing species. However, there is still concern regarding the secondary pollution that might occur in deciduous trees [208]. Even though woody biomass with high concentrations of Hg can be harvested and removed, the leaves are also rich in Hg. These require careful disposal too, otherwise they will fall and decompose, returning the Hg they contain to the soil.

Valuable knowledge has been acquired by engineering plants for Hg resistance, enabling important steps to be taken towards achieving more successful phytoremediation methods. Phytovolatilization, alone or coupled with organic Hg detoxification, is effective in Hg removal from soil, without tissue accumulation, but it would be suitable only for large severely polluted coastal areas, where the volatilized Hg^0^ can be dispersed by currents and diluted by the atmosphere, before redeposition at safe concentrations [184]. However, in inland fields, it would require the engineering of sealed systems coupled to condensation components that would trap and recover all volatilized Hg to prevent its atmospheric dispersal. Phytoextraction using transgenic plants capable of degrading and reducing mercury at the root level, with subsequent shoot oxidation and storage, is a preferable option for small areas, irrespective of their location, as Hg-loaded aerial parts are harvestable. Such a strategy would also be ideal for wetlands, where anoxic environment microbiota shift speciation to MeHg, but this assumes the use of wetland transgenic plant species.

## 6. Challenges and Perspectives for Hg Phytoremediation

Phytoremediation strategies for the decontamination of soils have long been considered more environmentally friendly, less costly and necessitating lower maintenance than engineering-based technologies. They can also be applied to large areas. Phytoremediation of Hg-contaminated sites has been aimed at either extracting, stabilizing or volatilising the metal. However, for phytoremediation of Hg-contaminated soils to become a reliable solution several problems need to be overcome. An important impediment is the high mobility of Hg between the environmental compartments, due to its ease of speciation. Ionic Hg from soil is frequently reduced to Hg^0^, especially by microorganisms, and then emitted into the air. Once in the air, Hg^0^ is easily dispersed and returned to the soil and plants by deposition, potentially leading to contamination of wider areas, albeit at lower concentrations. Plants exposed to aerial Hg contamination accumulate significant amounts of the metal that are linearly correlated with the concentration of Hg in the air [209,210,211]. High biomass vegetation such as willow, planted around the contaminated sites, can act as a buffer trapping the emitted Hg in its foliage, and thus reduce its dispersal. Indeed the leaves of *Salix* spp. and other deciduous species return annually, with the mercury they contain, to the soil. However, some of this can be uptaken and immobilized within roots. Nevertheless, (phyto)volatilisation of Hg should be carefully considered, and as far as possible efforts should be taken to capture gaseous Hg.

Phytostabilization of Hg-contaminated soils is a practical remediation solution because it mainly sequesters Hg within the roots of plants. It can be successfully applied to heavily contaminated areas that can not be used for agriculture. It requires plant species with a large and extensive root system that can tolerate high concentrations of Hg and restrict Hg translocation to aboveground tissues. Good progress has been made in establishing practical programmes for phytostabilisation, with several crops and plant species native to Hg contaminated soils already investigated. Many plant species that could be used for phytostabilisation might also be safe for seed-consuming birds, as Hg concentration detected in *N. tabacum* and *O. sativa* seeds was under the minimum permissible level [195]. Perennial species with secondary growth that accumulate mass and take up significant Hg quantities over an extended period are also likely to be suitable candidates for phytostabilization. As deciduous trees might contribute to secondary contamination *via* decomposition of their leaves each year, evergreen trees would be worth further investigation. Moreover, even though not applicable for crop production, Hg phytostabilization would produce biomass that, if properly treated, might provide resources to several industries

Phytoextraction of Hg would be the preferred solution especially for extensive, but only moderately contaminated areas, which might eventually be returned to agricultural use. Efficient phytoextraction would require identification of plant species able to uptake significant amounts of Hg, and further translocate and sequester it in aboveground tissues. For this purpose, identifying Hg (hyper)accumulator plant species is of utmost importance, since most plant species only have limited Hg buffering capacity. Furthermore, the Hg in soil must be in a bioavailable form. Interactions with appropriate microorganisms can aid in Hg acquisition and even secrete compounds that can augment plant growth. The most beneficial interactions between selected plant species and microorganisms still need further determination. Additionally, mechanised systems for planting and harvesting plant material, and systems for either safe disposal or further processing of contaminated plant material need to be developed.

Importantly, legal environmental and research funding entities need to understand that there is no universal solution for remediating Hg-contaminated sites. The soil properties, the bioavailability of Hg, the interactions between plants and microorganisms and the climate and the environmental conditions will be unique for each site. Use of plant and microorganism species that are not native to a particular site, can lead to undesirable species invasions, creating additional environmental problems. Therefore, use of plants and microorganisms that are pre-adapted to the conditions of particular areas is the ideal to be aspired to. Studies should also focus on identifying the molecular mechanisms of Hg uptake, distribution and fate in plant species, especially in those identified as potential (hyper)accumulators. Both phytostabilization and phytosequestration would benefit from integration of genes coding for proteins involved in Hg uptake, transport or sequestration under root- or tissue-specific promoters, and would enhance phytoremediation success over shorter time scales. More research is needed into the molecular interactions between plants and microorganisms. Omics analysis both in plants and microorganisms into their interactions would allow new useful genes and proteins to be identified, and would shed light on these interactions. The knowledge gained would result in the opening of further avenues for using and benefiting from these interactions. Finally, the logistics for efficient implementation of phytoremediation procedures and for recycling or safe disposal of the plant material involved need careful consideration.

## 7. Conclusions

Reclamation of Hg contaminated sites is of current interest, as significant areas of land might become productive if remediated. Phytoremediation of Hg-contaminated soils is an emerging strategy because plants handle contaminants without affecting the topsoil, thus preserving, or even improving its fertility through root exudation. Moreover, phytoremediation of sites can provide a low cost and low maintenance solution. To date, no Hg-hyperaccumulator species has been identified although significant progress has been made in identifying plant species that can grow on Hg-contaminated soil. Further assessment of plant species’ capacity to accumulate and translocate Hg when grown alone or in combination with microorganisms on contaminated sites is still necessary. Detailed research is needed to understand the molecular mechanisms underlying the uptake, accumulation, and sequestration of Hg in plants, and the interactions between plants and their associated microorganisms. Current knowledge of the roles of different microorganism communities in assisting Hg phytoremediation is limited. We suggest that thorough evaluation of the spatial and temporal distribution of plant-associated microbiomes in Hg-contaminated soils, and the distinctive niches they inhabit throughout plants’ developmental stages, would significantly increase understanding of their complex interactions, leading to the phytoremediation concept developing from its currently promising status into a reliable and valuable asset.

## Figures and Tables

**Table 1:** Summary of research on Hg phytoremediation potential and toxicity of Hg promising (hyper)accumulator species, wide-range heavy metal accumulator, and crop plants. * (Growth parameters recorded in regard to the control treatments).

Type	Plant sp.	Growth Conditions	Phytotoxic Concentration	Growth Parameters (Phytotox. Conc.) *	Hg Accumulation (BAF, BCF and TF)	References
potential Hg (hyper)accumulator native species	*Vigna unguiculata* L. Walp	Soil pots—3 m old ecotypes: 1. native genotype 2. commercial line L-019 3. commercial line L-042	5 and 8 mg kg^−1^ Hg(NO_3_)_2_ (added to 0.2 mg Hg kg^−1^ contaminated soil)	Negligible biomass decrease with ^ Hg	root > leaf > stem; BCF < 1 (all genotypes); BAF_stem/soil_ < 0.5, BAF_seed/soil_ < 0.5; 1. TF < 1 for native genotype 2. TF~1.5 (for 0.2 mg Hg kg^−1^ dw) for both commercial lines	[102]
	*Phragmites australis*	Plant samples were taken from gold mine contaminated wetland (wet and dry season)	-	-	root[Hg]—806 µg kg^−1^ dw stem[Hg]_—_495 µg kg^−1^ dw leaves[Hg]—833 µg kg^−1^ dw BAF—0.73/0.22 TF—0.57/1.99	[55]
	*Cyperus eragrostis*				BAF—0.22/0.35 TF—1.99/3/60	
	*Datura stramonium*				BAF—0.20/0.61 TF—4.26/8.30	
	*Panicum coloratum*				BAF—0.11/0.13 TF—3.70/10.94	
	*Persicaria lapathifolia*				BAF—0.11/0.20 TF—3.10/3.07	
	*Melilotus alba*				BAF—0.13/0.21 TF—0.54/0.60	
	*Lathyrus pratensis*	Aerial parts of plants growing in the area of an abandoned gold mine in the Czech Republic were collected (0.207–15.0 mg total Hg kg^−1^ soil)	-	-	Shoot[Hg]—0.108 mg kg^−1^ dw	[54]
	*Epipactis* sp.				Shoot[Hg]—0.152 mg kg^−1^ dw	
	*Axonopus compressus*	Plant samples were taken from soil contaminated by artisanal small-scale gold mines (arbuscular mycorrhizal fungi (AMF) colonization was aslo determined	-	-	root[Hg]—0.15 mg kg^−1^ dw shoot[Hg]_—_0.33 mg kg^−1^ dw BAF_root/leaves_—0.03/0.06 TF—2.16	[103]
	*Erato polymnioides*				root[Hg]—3.56 mg kg^−1^ dw shoot[Hg]—1.48 mg kg^−1^ dw BAF_root_—0.80; TF—0.42	
	*Miconia zamorensis*				root[Hg]—2.06 mg kg^−1^ dw shoot[Hg]_—_0.98 mg kg^−1^ dw BAF_root_—0.47; TF—0.47	
	*Cyrtomium macrophyllum*	60 d old seedlings from uncontaminated sites (grown 1st hydroponically) 1. 225.73 mg total Hg kg^−1^ soil or 2. 0, 5, 10, 20, 50, 100, 200, 500 and 1000 mg HgCl_2_ kg^−1^ soil	500 and 1000 mg kg^−1^ HgCl_2_	20.2% biomass reduction	1. shoot[Hg]—36.44 mg kg^−1^ dw root[Hg]—13.90 mg kg^−1^ dw BCF—0.061; TF—2.62 2. for treatments up to 200 mg kg^−1^: BCF > 1; TF > 1	[53]
	*Manihot esculenta* Crantz	1. soil pots with mixtures of mine tailings and biosolids; 4 w old cuttings ( 11.67 mg total Hg kg^−1^ mine tailings); 2. hydroponic solution with 50 or 100 µM HgCl_2_; 5 w old plants	mixtures with 50, 75, or 100% mine tailings	significant root biomass decrease	1. Hg not determined in plants 2. root[Hg]—6.836 and 12.13 g kg^−1^ dw (50 and 100 µM Hg)	[104]
	*Dillenia suffruticosa*	Plants were cultivated on 2 ex-gold mine tailings areas: (i) tailings site where last mining activity was 2 years prior (0.5 mg Hg kg^−1^) (ii) tailings site where last mining activity was 10 years prior (0.02 mg Hg kg^−1^)	none observed	no significant decrease in plant growth (height and diameter)	BCF—15.5; TF—3.0	[50]
	*Vitex pinnata*				BCF—40; TF—0.6	
	*Archidendron pauciflorum*				BCF—11.0; TF—0.1	
	*Anacardium occidentale*				BCF—6.5; TF—0.3	
	*Shorea leprosula*				BCF—7.5; TF—0.5	
	*Alstonia scholaris*				BCF—45.0; TF—1.3	
	*Hevea brasiliensis*				BCF—13.5; TF—0.1	
	*Alyssum saxatile* L.	Plant samples were collected from 41 sites in an active mining district in Western Turkey (mean 6.609 µg Hg kg^−1^ soil)	-	-	root[Hg]/soil[Hg]—0.10 shoot[Hg]/soil[Hg]—0.04 Mean TF—0.85	[52]
	*Anchusa arvensis* L.				root[Hg]/soil[Hg]—0.06 shoot[Hg]/soil[Hg]—0.06 Mean TF—1.03	
	*Centaurea cyanus* L.				root[Hg]/soil[Hg] < 0.5 shoot[Hg]/soil[Hg] < 0.5 Mean TF > 1	
	*Cynoglossum officinale*				root[Hg]/soil[Hg] < 1 shoot[Hg]/soil[Hg] < 1 Mean TF < 1	
	*Glaucium flavum*				root[Hg]/soil[Hg]—0.09 shoot[Hg]/soil[Hg]—0.02 Mean TF—0.25	
	*Isatis* sp. L.				root[Hg]/soil[Hg]—0.02 shoot[Hg]/soil[Hg]—0.02 Mean TF—0.63	
	*Onosma* sp.				root[Hg]/soil[Hg] < 0.5 shoot[Hg]/soil[Hg] < 0.5 Mean TF > 1	
	*Phlomis* sp.				root[Hg]/soil[Hg]—0.21 shoot[Hg]/soil[Hg]—0.56 Mean TF—2.05	
	*Silene compacta*				root[Hg]/soil[Hg] < 0.5 shoot[Hg]/soil[Hg] < 0.5 Mean TF—1.66	
	*Tripleurospermum maritimum*				root[Hg]/soil[Hg]—0.02 shoot[Hg]/soil[Hg]—0.01 Mean TF—0.59	
	*Verbascum thapsus* L.				root[Hg]/soil[Hg]—0.03 shoot[Hg]/soil[Hg]—0.06 Mean TF—2.47	
	*Sesbania grandiflora*	17 d old seedlings in hydroponic solution	50 and 60 mg L^−1^ HgCl_2_	56% growth decrease 19% biomass reduction (60 mg Hg L^−1^)	mostly in roots; TF—low.	[91]
	*Jatropha curcas*	Pots with Hg-contaminated soil (1.76 mg kg^−1^) spiked with 1, 5 or 10 mg Hg(NO_3_)_2_ kg^−1^; 1, 2, 3 or 4 m old seedlings (seeds of plants from uncontaminated soil)	none observed	-	plant[Hg]—max. 7.25 mg kg^−1^ dw (for 10 mg Hg kg^−1^ soil) BCF—good, with increased exposure (4th month); TF~1 (after 2 months, then decreased)	[105]
	*Lepidium sativum* L.	Soil pots (spiked with 10 or 100 mg HgCl_2_ kg^-1^ dw) with/without different fractions of uncontaminated compost; 10 d seedlings	(a) 10 and 100 mg kg^−1^ HgCl_2_; (b) none observed for compost amended soil	(a) 27% decrease in shoot length; 53% decrease in root (10 mg Hg kg^−1^)	mostly in roots; add. compost—^ accumulation; BCF—high for 10 mg Hg kg^−1^ dw in 2/1 compost	[106]
	*Flueggea tinctoria* (L.) G.L. Webster	Aerial plant parts were collected from a riparian area in the mining district of Almadén (122—385 mg total Hg kg^−1^ soil)	-	-	BCF—5.9	[49]
	*Tamarix canariensis* Willd.				BCF—10.72	
	*Nerium oleander* L.				BCF—6.2	
	*Typha domingensis* Pers.				BCF—4.3	
	*Phragmites australis* Cav.				BCF—32.2	
	*Atriplex conodocarpa*	25 seeds/species were sown in pots with Hg spiked potting mix (17.3 mg Hg kg^−1^ soil)	no phytotoxic symptoms were observed	Biomass, leaf area and number remained unchanged (in regards to unspiked soil)	shoot[Hg]—1.09 mg kg^−1^ dw translocation %—19%	[107]
	*Australodanthonia caespitose*				shoot[Hg]—1.20 mg kg^−1^ dw translocation—15.9%	
	*Chilopsis linearis*	2 w old seedlings in Hoagland solution	50, 100, 200 µM (CH_3_COO)_2_Hg	49% decrease in root length	root[Hg]—^ with Hg conc. TF—low	[108]
	*Medicago sativa*	4 d old seedlings in 1/4 Hoagland solution	20 µM HgCl_2_	54% decrease in root biomass	-	[88]
	*Eichornia crassipes*	30 d old plants in spring water tanks (0, 0.5, 2 mg L^−1^ HgSO_4_)	-	-	root[Hg]—26.2 mg kg^−1^ dw (for 2 mg Hg L^−1^)	[101]
	*Pistia stratiotes*				root[Hg]—83.2 mg kg^−1^ dw	
	*Scirpus tabernaemontani*				root[Hg]—3.88 mg kg^−1^ dw	
	*Colocasia esculenta*				root[Hg]—6.99 mg kg^−1^ dw	
	*Sesbania drummondii*	15 d old seedlings in 1/2 Hoagland solution	50 and 100 mg L^−1^ HgCl_2_	36.8% biomass reduction (100 mg Hg L^−1^)	root[Hg] > shoot[Hg]	[89]
	*Rumex induratus*	Field experiment; Whole plants were collected from sites with: 122.4 mg total Hg kg^−1^ dw (0.006% available Hg)	^-^		root[Hg]—8.3 mg kg^−1^ dw shoot[Hg]—7.3 mg kg^−1^ dw TF—0.96 Phytoextraction efficiency 12.9 g Hg ha^−1^ year^−1^	[109]
	*Marrubium vulgare*	550.1 mg total Hg kg^−1^ dw (0.032% available)			root[Hg]—67.2 mg kg^−1^ dw shoot[Hg]—23.0 mg kg^−1^ dw TF—0.34 Phytoextraction efficiency 27.6 g Hg ha^−1^ year^−1^	
	*Medicago sativa*	12 d old seedlings in a beaker-size hydroponic system	30 µM HgCl_2_	abrupt 30–40% growth inhibition (first 24 h)	-	[87]
	*Myriophylhum aquaticum* *Ludwigina palustris* *Mentha aquatica*	21 d old plants in water solution with hydroponic fertilizer	-	-	average removal efficiency—99.8% (all 3 plants); removal rate—0.0787–0.0002 mg Hg L^−1^ d^−1^	[100]
	*Nicotiana miersii*	5 w old plants in 1/4 Hoagland	1. 1.0 mg Hg^0^ m^3^ 2. 1.0 µg HgCl_2_ mL^−1^	1. Visible signs of stress 2. Inhibition of root and shoot	1. only in shoots 2. mostly in roots	[110]
broad-spectrum heavy metal (hyper)accumulator species	*Brassica juncea* Long-standing and Florida Broad Leaf cultivars	2 and 4 w old plants grown hydroponically	1.96, 4.11, 12.2, and 16.7 mg L^−1^ Hg(NO_3_)_2_	25% biomass decrease	BCF_root_—750–1100; BCF_shoots_—82–104; roots[Hg]/shoot[Hg]—8–100	[111]
	*Brassica juncea*	36 d old seedlings grown hydroponically	5 and 10 mg L^−1^ HgCl_2_	5.1-fold reduced transpiration rates	BCF_root_—100–270; BCF_shoot_—0.31–1.07; shoots[Hg]/root[Hg]–0.3–0.76	[112]
crop plant species	*Hordeum vulgare*	Soil pots—3 soil compositions: 1. 8.35 mg HgCl_2_ kg^−1^ dw; 2. 32.16 mg total Hg kg^−1^ dw; 3. 32.16 mg total Hg kg^−1^ dw + 1 mg HgCl_2_ kg^−1^; 150 d old plants	-	-	1. shoot[Hg]—1.51–5.13 mg kg^−1^ dw; (*L. esculenta* and *L. albus* the highest); 2. shoot[Hg]—0.16–1.13 mg kg^−1^ dw; 3. shoot[Hg]—6× *L. albus*, 5× *C. aretinum*, 3.5× *H. vulgare* and *L. esculenta* (* regards to 2nd treatment)	[113]
	*Lupinus albus*					
	*Lens esculenta*					
	*Cicer aretinum*					
	*Cucumis sativus*	10 and 15 d old seedlings in 10% MS media	250–500 µM HgCl_2_	96% root length reduction (10 d old seedlings) 98% root length reduction (15 d old seedlings)	root[Hg]—7-fold and 5.6-fold > cotyledons (after 10 and 15 d)	[114]
	*Oryza sativa*	3 w old seedlings in Long Ashton modified nutrient solution	0.5 mg L^−1^ HgCl_2_	50% shoot biomass reduction	root[Hg] 2× > shoot[Hg] BCF~1900 (for higher Hg conc.)	[115]
	*Lycopersicon esculentum*	30 d old seedlings in modified Hoagland	50 µM HgCl_2_	suppressed biomass production (roots and shoots)	root[Hg]—27-fold > shoot; uptake ^ linearly with concentration	[90]
	*Pisum sativum*	seedlings in solution culture	5 and 10 mg L^−1^ HgCl_2_ or ^203^HgCl_2_	growth inhibition: 50% shoot and root length decrease (10 mg Hg L^−1^)	mostly in roots; linearly increase with [Hg]; TF—low	[116]
	*Mentha spicata*	cuttings in solution culture				

BCF = Bioconcentration Factor (plant[Hg]/corresponding soil or media[Hg]; depending on study, plant[Hg] can refer to root[Hg]); BAF = Bioaccumulation Factor (shoot[Hg]/corresponding soil or media[Hg]); TF = Translocation Factor (shoot[Hg]/root[Hg]); ^ = increase; d = days; w = weeks; m=months; [Hg] = Hg concentration.
